# Nanochitin whisker enhances insecticidal activity of chemical pesticide for pest insect control and toxicity

**DOI:** 10.1186/s12951-021-00792-w

**Published:** 2021-02-16

**Authors:** Zhenya Li, Hezhong Wang, Shiheng An, Xinming Yin

**Affiliations:** 1 Department of Entomology, College of Plant Protection, Henan Agricultural University, 450002 Zhengzhou, China; 2 Department of Pesticide Science, College of Plant Protection, Henan Agricultural University, 450002 Zhengzhou, China; 3 Department of Pesticide Science/Nano Agro Center, College of Plant Protection, Henan Agricultural University, 450002 Zhengzhou, China

**Keywords:** Nanochitin whisker, Insecticidal activity, Synergy, Mortality, Wheat aphids, Toxicity

## Abstract

**Background:**

Nanomaterials in plant protection promise many benefits over conventional pesticide products. Nano-enabled pesticides may alter the functionality or risk profile of active ingredients. Cationic nanochitin whiskers (NC) possess strong biological activity against wheat aphids. However, toxicity and synergistic effects of NC with chemical pesticides against pest insects has not been systemically reported. This study investigated the insecticidal enhancement by NC with Omethoate (40% EC), Imidacloprid (10% WP), and Acetamiprid (40% WG) for pest control using wheat aphid as piercing-sucking mouthparts insect. Fluorescein isothiocyanate labelled NC was used to monitor the uptake and transportation pathway of NC inside the target insects. Toxicity of NC was tested with Sprague-Dawley (SD) rat. Our findings provide a theoretical basis for future application of NC in plant protection against pest insects.

**Results:**

NCs synthesized by acidic hydrolysis were rod-like nanoparticles in a range of 50–150 nm in length and 30–50 nm in width, which examined by electron microscopy and dynamic light scattering methods. The charge density and zeta potential were about 63 mmol/kg and + 36.4 mV, respectively. By absorption and/or contact action of 30–50 mg/L of NC suspension, the corrected mortality of wheat aphids reached up to 80% or above after 12 h treatment, NC could be distributed through digestive system and relocated from mouth to other tissues inside the insect body. When associated with dilutions of conventional pesticides, the corrected mortality were significantly increased up to 95% or above. The dosage of the chemical pesticide and nanochitin in the mixtures (1:1 by volume) were all reduced to half. The acute oral toxicity Lethal Dose 50% (LD_50_) to SD rat is greater than 5000 mg/kg BW (body weight) in male and female, acute dermal toxicity LD_50_ is greater than 2000 mg/kg BW of NC.

**Conclusions:**

NC has a strong promotive effect on insecticidal effectiveness of chemical insecticides. It was easily absorbed by plant, transported and distributed from mouth to other tissues of the insects while sucking plant fluid. Low acute oral and dermal toxicity to SD rat indicated that it is safe to apply in agriculture and food industry. NCs has a great potential for water-based nanopesticide formulation to reduce chemical pesticide use for future agro-environmental sustainability.
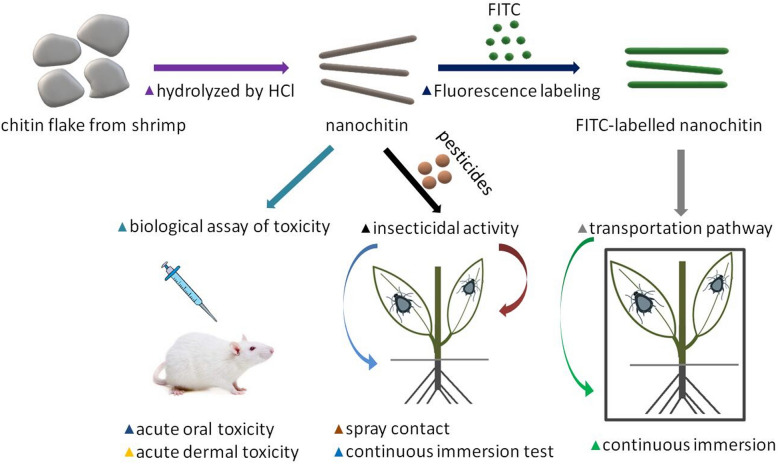

## Introduction

As a recognized cutting edge technology, nanotechnology has been applied across a wide range of areas, perhaps more recently in the natural sciences with significant interest in agriculture and food applications over the past decades [1, 2]. Since nanoscaled particles have an extremely small size, high surface to volume ratio and active surface area, their unique properties and superior functionalities greatly differ from those of micro-sized particles or fibers [3]. The advantages of the application of nanomaterials in agriculture are in particular to improve pesticides activity, enhance the yield and quality of crops, reduce agrochemicals usage and cost in plant protection, minimize nutrient losses in fertilization, and scale down the environmental pollutions [4, 5]. Nano–formulations of pesticides have been rapidly developed to replace classic pesticides since the millennium [6]. Nanoparticles may act as plant growth promotors, and/or enhancers for stress tolerance to harsh environment. Their functionality and efficacy not only depend on their physiochemical properties, but also application methods (foliar delivery, hydroponics, soil). Doses of application make great effects in effectiveness [7]. Liu et al.[8] proposed that nano-scaled formulation of pesticide provided a better spatial distribution of the pesticide and uniformity of coverage on leaf surfaces during enhancing the insecticidal efficiency. Similar as other nano-formulated products, excessive utilization of engineered inorganic, or manipulated nanoparticles in agriculture might also have an unknown consequences or co-contamination in soil, ground water, and food products, even potentially toxic to human and plants [2]. Therefore, alternatives of nanopesticide formulation with biomaterials have aroused great interest recently.

Chitin is a naturally polymer in the form of microfibrillar arrangements in fungal cell walls, crustacean shells, and insect exoskeletons, constituting of randomly distributed long - chain of N-acetyl-D-glucosamine (GlcNAc), β (1–4) linked residues of N-acetyl-2 amino-2-deoxy-D-glucose, and 2-amino-2-deoxy-D-glucose residues (GlcN) [9]. Native chitin polymer occurs in three forms including α-, β-, and γ-types crystallites, which depends on their biological origin [10]. Most natural chitin has α- type crystal structure with strong intermolecular hydrogen bonding, while β- type chitin is presented in squid pens and tubeworms [11]. Chitin is insoluble in water and most organic solvents due to its highly hydrophobic structure. These three forms of chitin are all stable after boiling in 5% KOH or NaOH, α-chitin is even more thermodynamically stable than β - chitin [12].

Chitosan is the most important derivative of chitin but it is referred only when the degree of deacetylation reaches circa 50% or higher, depending on the origin of the polymer, and becomes soluble in aqueous acidic solution [13]. Chitin and its derivatives have been widely applied in the food science, agriculture, cosmetics, wastewater treatment, and biomedical areas [14] due to its desirable biological properties, biocompatibility, biodegradibility, and low cytotoxicity to human and other animals [15–18]. Chitin and chitosan have been recognized as plant growth regulators, growth stimulants, and elicitors for the production of secondary metabolites acting as fertilizers, soil conditioning agents, plant disease control agents, antitranspirants, ripening retardants, and seed and fruit coatings [19–23]. Early studies showed that chitosan and chitosan-metal complexes ( e.g. chitosan-silver nanoparticle) had a potential to develop a new control tools against young instar populations of malaria mosquitoes [24]. But the insecticidal activity of chitosan and chitosan-metal complexes against cotton leafworm *Spodoptera littoralis* and oleander aphid *Aphis nerii* as sucking pest were related to molecular weight and degree of deacetylation of chitosan. Chitosan with molecular weights in the range of 2.27 × 10^5^ − 5.97 × 10^5^ g/mol showed the highest efficacy in pest control [25]. However, chitin and chitosan are mainly distinguished not only by their solubility in dilute aqueous acids, but also related to the degree of deacetylation, and the method of preparation as well [26, 27]. Water insolubility of chitin and chitosan is the key factor limiting their use in agriculture.

Nanochitin whisker (NC) has recently drawn a growing attention in agricultural and environmental application [28–33]. NC is generally synthesized chemically or enzymatically [34, 35]. Chitin is fairly stable under mild acidic and basic conditions. However, in a concentrated hydrochloride acid solution, deprotonated and demineralized chitin can be degraded and partial deacetylated from amorphous region of chitin microfibrils and resulted in nanocrystalline fragment, i.e. nanochitin particle or nanochitin whisker [36]. In acidic solution, some unacetylated amino-groups of the NC complexed with a proton to give NH_3_^+^ at the crystallite surfaces. The charged crystallites particles repel each other and form a stable colloidal suspension at a concentration less than 1% [37]. In other words, the protonation of amino group brings positively charged particle and yields polyelectrolyte in solution. The acid concentration, reaction temperature, and time are main factors influencing the products’ physicochemical properties including the particle size, surface charge, and its chemico-biological properties. On the other hand, ionic strength affects the stability and charge density of nanochitin in aqueous solution [38]. Generally, the particle size of chemical synthesized nanochitin varies in the range of 50 to few microns in length and 4–80 nm in width depending on the hydrolysis methods and different origins of chitin [12, 39].

The nanochitin whisker possesses strong biological activities, low toxicity in plant protection application [32, 40–44]. It has been reported that nanochitin particle surpasses reported biobased nanoparticles such as nanocellulose, alginate, and glucan. Its interface stability at ultralow concentrations and utilization potentialities has more advantages than other biobased nanoparticles in future nanopesticide formulation through self-assembled networks of short and long nanoparticles for oil/water interfacial super stabilization [1, 45]. Nanochitin has shown great potential in various biomedical, agriculture and food applications. A recent report demonstrated that nanochitin particles are useful for functional foods formulation as a nano-emulsifier because of their strong ability to slow down lipid digestion inside the human gastrointestinal track without toxicity to humans, animals, wildlife, or the environment [38]. Based a report of cytotoxicity assay, both nanochitin particle and fiber were non-toxic to epithelial-like and fibroblast-like cell lines [46]. Zhao et al. [47] also demonstrated that chitin whisker had good biocompatibility below the concentration of 2.5 mg/mL with low toxicity to model cells of mouse bone marrow mesenchymal stem cells (mBMSCs) and rat osteosarcoma cells (UMR-106) in vitro. In our previous study, nanochitin mixed with Imidacloprid 10% WP showed a significant control efficiency in wheat aphid [44]. We recently reported that nanochitin conjugated with abamectin, a biopesticide for insect pests control, had a great ability to control larva growth, enhancing mortality against 2 instar larvae of noctuid, and effectively reducing pesticides dosage by 50% [43]. However, the insecticidal activity and toxicity of NC has not been systemically explored at present. The hypothesis of this study is that NC can be absorbed and transported inside a biological system easily due to its small size, cationic nature. Water based nanochitin suspension is a good candidate in nanopesticide formulation to enhance insecticidal activity and safer to use in agriculture. The objectives of this study were to assess the synergistic effects of NC with chemical pesticides on piercing-sucking mouthparts insect control using wheat aphid as a model insect, and monitor the transportation pathway of NC inside insect bodies using fluorescent-labelled nanoparticles. To ensure a safer application, we studied the toxicity of NC against Sprague–Dawley (SD) rat as a model animal. The aim of this study is to provide innovative solutions in nanopesticide formulations and application in the pest control for future agriculture sustainability.

## Materials and methods

### Materials

Chitin (from shrimp shells practical grade, powder) was purchased from Sigma Aldrich (Sigma-Aldrich Co. LLC.), hydrochloric acid (36–38%) was purchased from Shuangshuang Chemical Company (Yantai, Shandong, China), anhydrous ethanol was purchased from Fuyu Chemical Company (Tianjin, China), fluorescein isothiocyanate (FITC) was purchased from Sangon Biotech (Sangon Biotech Co., Ltd. Shanghai, China), standard HCl solution (certified, 0.1 M), NaOH solution (certified, 0.1 and 1 M), acetic acid (glacial, certified plus), and NaCl (certified) were purchased from Thermo Fisher Scientific Co., Ltd. (Shanghai, China). Omethoate (40% EC) was purchased from Tianyi Agrochemical Crop (Zhejiang, China). Imidacloprid (10% WP) was purchase from Hailir Pesticides and Chemicals Group (Shandong, China). Acetamiprid (40%WG) was purchased from Shuang Xing Pesticide Co., LTD (Shandong, China).

Wheat plants were planted in the Insect Physiology Laboratory at Henan Agricultural University, China. The model aphids (*Rhopalosiphum padi*, Hemiptera: Aphididae) were bred in the Plant Growth Chamber for at least 30 generations in Insect Physiology and Biochemistry Laboratory of Henan Agricultural University. Adult male and nulliparous, non-pregnant female *Sprague Dawley* (SD) rats were obtained from Henan Laboratory Animal Center (Zhengzhou, China) and bred under specific-pathogen-free conditions for Biological assay of toxicity.

The water used in the experiments was deionized water from a Millipore Direct-Q 5 ultrapure water system (resistivity at 25 ^o^C: 18.2 MΩ cm). All other chemicals were analytical grade.

### Preparation of nanochitin

Nanochitin was prepared according to the method described by Xue et al. [32] with some minor modifications. In brief, shrimp chitin powder was hydrolyzed in 3 M of HCl solution at a reaction ratio of chitin to HCl of 1 g: 400 mL for 90 min at 90 ℃ under an overhead stirring at 150 rpm to digest the amorphous regions of chitin. The reaction was repeated three times at same condition. After each hydrolysis, the reaction was stopped by cooling the reaction flask in an ice-water bath, the treated chitin was recovered by centrifugation at 9000 pm for 15 min under cooling (4 ^o^C), and same amount of fresh reagent was then added to the reaction system to finish the next round reaction. Following the final step of hydrolysis, the reaction was stopped by 10-fold dilution of the reaction medium with pre-cooled deionized water. The product was collected by centrifugation for 15 min at 9000 rpm under cooling (4 ^o^C) and redispersed in deionized water after discarding of the supernatant. For removal of the remaining acid, the final product was dialyzed against deionized water with regenerated cellulose dialysis tubing (Spectra/Por4, Spectrum Laboratories, MWCO 12–14 kDa) until the pH of the dialysis water stayed constant. The obtained suspension was sonicated under ice-bath cooling for 15 min at 35% output with a 500 W ultrasonic processor (Sonics & Materials, model VC-505). Finally, the suspension was filtered through a 1.0 µm and then 0.45 µm PVDF syringe filter. The stock suspension of nanochitin was stored at 4 ^o^C for future characterization.

### Characterization of nanochitin

The concentration of nanochitin suspension (NCs) was measured by oven-dry for 4 h at 80 ^o^C. Briefly, 1.0 mL of NCs was deposited and weighted before and after drying for 4 h in an oven at 80 ℃. The concentration was calculated based on the weight loss of NCs, the measurement was conducted in triplicate. The concentration in this study was generally in the range of 0.3–0.6% (w/v).

The morphology of the nanochitin particles was analyzed by field-emission scanning electron microscopy (FE-SEM) and transmission electron microscopy (TEM) respectively. For FE-SEM, a drop of 10 µL of NCs (0.015% w/v) was deposited onto a conductive double-sided carbon adhesive tape (SPI Supplies and Structure Probe, Inc.) mounted onto a standard SEM stub and allowed to dry under ambient conditions. Prior to imaging, the SEM samples were coated with platinum. Images were recorded with a JSM-6490LV scanning electron microscope at an accelerating voltage of 10.0 kV and a working distance of 9.5 mm. About 10–15 particles on the image were randomly selected to measure the geometric sizes (length and width) using automated software (JEOL Scanning Electron Microscope software, 6490 SEM). For TEM, a drop of the NCs (0.075% wt/v) was deposited on carbon-coated copper grid (200 meshes). The excess liquid was absorbed by filter paper, and 1 drop of 2% uranyl acetate negative stain was added before drying. The excess solution was blotted with a filter paper and allowed to dry naturally by evaporation. The sample grid was observed using a JEOL electron microscope (JEM 2100) with an accelerating voltage of 80 kV. The images were acquired with a 4k CMOS camera.

For the particle size and zeta potential measurement, a 0.1% (W/V) of NCs was diluted from stock solution by deionized water without adjustment of the pH and ionic strength. The effective particle size and zeta potential of nanochitin particles were measured based on dynamic light scattering (DLS) technique by 90 Plus Zeta (Brookhaven Inc.) at 25 ^o^C. Measurements were performed in triplicate at 25 ^o^C.

The amino group density of the nanochitin was measured in triplicate by conductometric titration with FE30 conductivity meter with an LE703 conductivity probe. The initial pH of the suspension was adjusted with a standard HCl solution (0.1 M), having an ionic strength of 0.1 M. A standard NaOH solution (0.01 M) was added in drop-wise under stirring to 20 mL of a 0.27% (w/v) of NCs, at every 10th drop, the conductivity of the NCs was recorded. The amino group density was calculated from the titrant volume between the two equivalence points. All measurements were performed in triplicate.

### Preparation of fluorescent‐labelled nanochitin

Fluorescent-labelled nanochitin particles were prepared following method described by Zhao et al. [48] with some modification. In brief, about 200 mg (0.33% mg/ml, 60 mL) of purified nanochitin was re-dispersed in 60 mL of 1% (v/v) acetic acid solution followed by the addition of 0.1 M NaOH solution to adjust pH to 6.2. FITC was dissolved in methanol at 10.0 mg/mL concentration, and 3 mL of FITC solution was slowly added to the NCs. The labelling ratio of the amino group in total glucosamine unit was controlled with a final concentration of FITC in the reaction medium. The reaction between the isothiocyanate group of FITC and the amino group of the D-glucosamine residue was allowed to proceed for 4 hours in the dark at room temperature. FITC-labelled nanochitin particles (FNC) were centrifuged at 12,000 rpm for 15 min and washed with methanol extensively until there was complete absence of free FITC fluorescence signal in the washing medium, which was detected using UV-vis spectrophotometer (UV-2000, UNICO Instrument Co., Ltd., Shanghai), and the values for peak fluorescence intensity was determined at 490 nm. The standard absorbance of FITC was calibrated using dilutions of FITC in methanol. Fluorescent intensity of FNC was examined by fluorescent microscopy, which excitation and emission spectrum peak wavelength of FITC was at approximately 495 nm and 519 nm respectively. The labelling efficiency was approximately one FITC molecule per 70 of D-glucosamine residues of nanochitin. Typical reaction yield was 85%.

### Insecticidal activity of nanochitin against pest insect

#### Spray contact

This study was carried out using wheat aphid as piercing-sucking mouthparts insect. The insecticidal activity assay followed the Guideline for Laboratory Bioassay of Pesticides (Ministry of Agriculture of the People’s Republic of China, NY/T 1154.9–2008). In brief, wheat aphid *R. padi* was obtained after at least 30 generation breeding from the Insect Physiology and Biochemistry Laboratory of Henan Agricultural University, 15 of healthy and active aphids were selected for a standard screening toxicity test by spraying contact method with difference concentration of NCs and dilutions of three insecticides 40% Omethoate (EC), 40% Acetamiprid (WG), and 10% Imidacloprid (WP), as well as their mixtures. The selected aphids were carefully inoculated onto the leaf of wheat plant using a soft brush. NCs with concentrations of 10 mg/L, 30 mg/L, and 50 mg/L were diluted from a stock suspension (0.38% w/v) with deionized water, respectively. Recommended application concentrations of 1500-fold solution (1500×, 1 in 1500 parts dilution) of 40% Omethoate (EC), 16,000-fold solution (16,000×, 1 in 16,000 parts dilution) of 40% Acetamiprid (WG), and 3000-fold solution (3000×, 1 in 3000 parts dilution) of 10% Imidacloprid (WP) were prepared by diluting 40% Omethoate (EC), 40% Acetamiprid (WG), and 10% Imidacloprid (WP) with tap water, respectively. The control efficiency was compared among the treatments of the dilutions of NCs, 1500× solution of 40% Omethoate (EC), and 16,000× solution of 40% Acetamiprid (WP), as well as their mixtures with NCs, respectively. The 1500× solution of 40% Omethoate served as a control. Aphids were fed under the condition of 24–26 ℃, 60% − 80% of relative humidity, the photoperiod L: D = 16:8. The number of aphids alive was checked at interval times after treatment. To define aliveness of aphid after treatment, a soft slender brush was adopted to gently touch the body of aphid, those of irresponsive bodies were counted as dead, and corrected death rate and the mortality were then calculated. All treatments were repeated three times.

### Continuous immersion test

Continuous immersion test was carried out by leaf-dipping method following the Guideline for Laboratory Bioassay of Pesticides—Ministry of Agriculture of the People’s Republic of China NY/T 1154.4–2008. Nanochitin with concentration of 10 mg/L, 30 mg/L, and 50 mg/L were also used to evaluate the control efficiency and synergistic effects on mortality of aphid when combined with conventional pesticides in this study. The 1500× dilution of 40% Omethoate served as a control. The control efficiency was compared with different concentrations of NCs and the mixtures with 3000× dilution of 10% Imidacloprid (WP), 1500× dilution of 40% Omethoate (EC), and 16,000× dilution of 40% Acetamiprid (WP), respectively. For the treatment, the healthy wheat plants with roots were selected, fully expanded true leaves from wheat plants were dipped in NCs for 30 s and air-dried for 30–60 min. The treated leaves were then placed on filter papers in Petri dishes (three petri dishes per concentration). Once treated leaves had relatively dried, 15 of healthy adult aphids were inoculated to each treatment. The treatments were kept in a constant environment room maintained at 26 ^o^C ± 2 ^o^C, 65 ± 5% RH, with 12 h: 12 h (light: dark) photoperiods. Mortality was recorded at 24, 48, 72, and 96 h after treatment under a microscope. Aphids unable to move and showing symptoms of poisoning were counted as death. Experiments were carried out in triplicate.

The model used for calculating the mortality was based on the Guideline for Laboratory Bioassay of Pesticides –Ministry of Agriculture of the People’s Republic of China NY/T 1154.4–2008, corrected mortality were calculated according to following equation:

$$\text{Corrected}\;\text{mortality}\;(\%)\;=\;(P-P_0)/(1-P_0)\;\times\;100$$

where: P is the percentage of mortality of treated insects, P_0_ is the percentage of mortality of insects in the untreated control.

### Biological assay of toxicity

The biological assay was conducted by School of Public Health of Zhengzhou University (Zhengzhou, China) following the Toxicological Test Methods of Pesticides for Registration-State Standard of the People’s Republic of China-GB15670-1995.

### Acute oral toxicity

Acute oral toxicity of nanochitin was evaluated using *Sprague Dawley* (SD) rats at School of Public Health of Zhengzhou University (Zhengzhou, China), based on Good Laboratory Practice (GLP). This experiment was conducted in accordance with the authorized guideline for care and use of the laboratory animals. Five male and five female SD rats in specific pathogen-free (SPF) grade were selected and kept on an ordinary diet and were breeding at ambient conditions (21–25 ^o^C, relative humidity 50–70%). The rats were divided into two groups, 5 rats each group with an average body weight of 189–201 g. Permit limited tests method was used, 5000 mg/kg body weight (BW) of the test animal was administered orally by gavage to 5 rats that have been overnight fasted, oral administration of NCs (1.0 mL/100 g BW) in one oral gavage dose for 2 weeks. After 2 weeks of observation, animal weight, symptoms of poisoning, and death were recorded. The death of the animal for gross pathological examination was carried out at the end of the test. The value of LD_50_ was the average of each treatment.

### Acute dermal toxicity

The tested SD rats and the method were same as described above. For dermal toxicity test, permit limited tests of 2000 mg/kg•BW of the test animals was used. Before test, the hair on the animal back was shaved and the damage of the skin was observed for 1d. The test was carried out by placing NCs on an area of 4.5 cm^2^ where the animal’s skin has been shaved. The applied area was then covered with sterilized gauze. After 4 h adsorption, the treated area was washed with warm water and then observed the changes for 14 days, and all gross pathological changes were recorded. Necropsy of all animals was carried out and all gross pathological changes were recorded.

### Statistical analysis

The data represents the means for each treatment and compared statistically with a one-way analysis of variance LSD (ANOVA, α = 0.05). All measurements were carried out in triplicate. Standard deviation was used to specify variability among triplicates. Differences at p < 0.05 or p < 0.01 were considered to be statistically significant. Data was analyzed using SPSS 22.0 software for Windows (IBM, New York, NY, USA).

## Results and discussion

### Characterization of synthesized nanoparticles

**Fig. 1:**
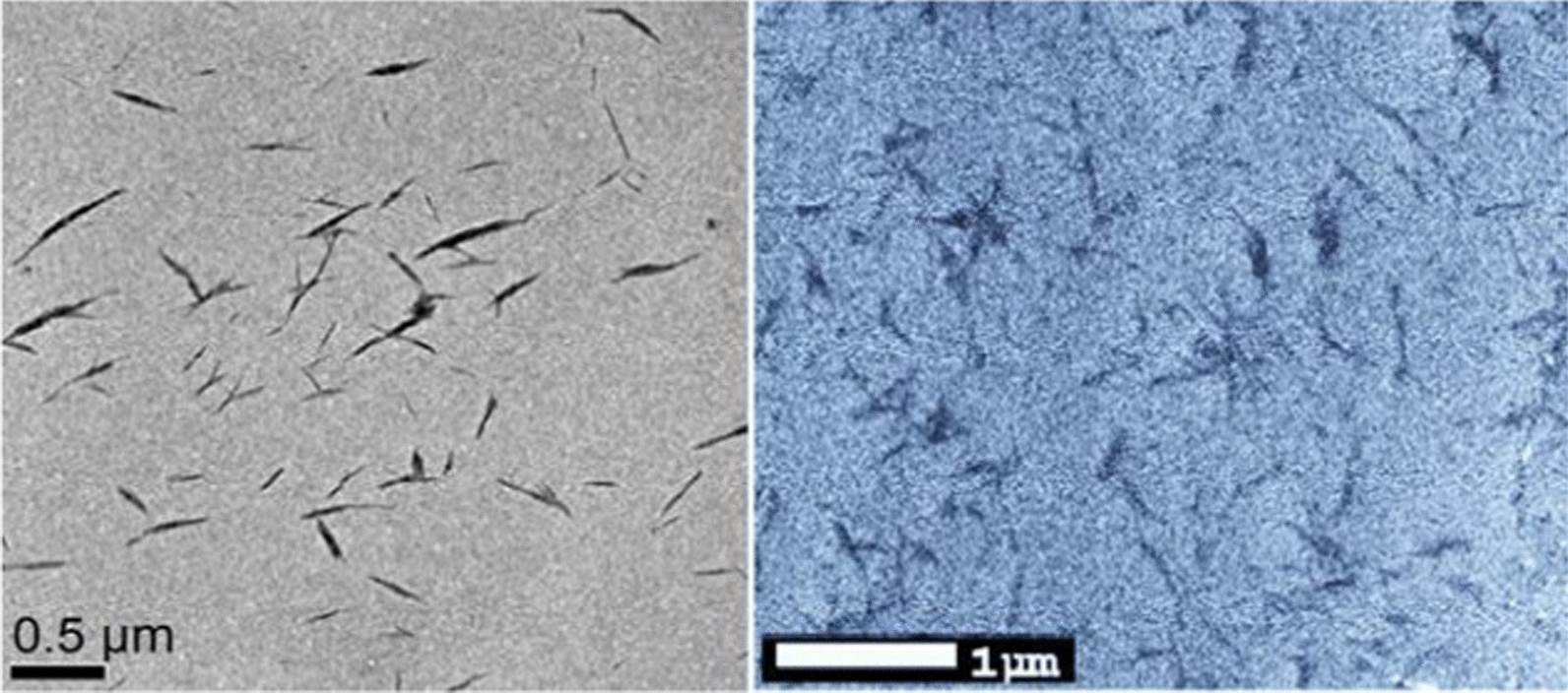
Electron microscopy images of chemically hydrolyzed nanochitin particles. Left-TEM image (sale bar, 0.5 µm), right-FE-SEM image (sale bar, 1.0 µm)

The morphology of nanochitin particle was studied by TEM and FE-SEM respectively. Similar morphology of the particles was observed on both SEM image and TEM image (Fig. 1). The length of the particles was in the range of 50–150 nm, the width was 15–50 nm, which makes a great agreement with previous reports [32].

**Fig. 2:**
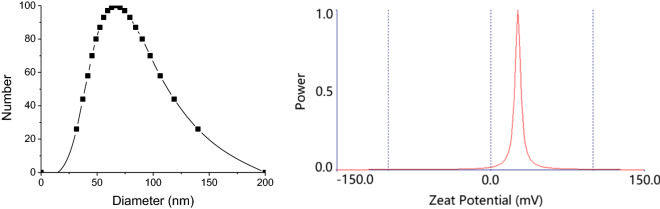
The size (left) and zeta potential (right) distributions of nanochitin particles examined by dynamic light scattering

DLS measurements are commonly technique used to determine the true state of particles in media [32]. The determination of size, zeta potential, and polydispersity (PDI) parameters are defined in the ISO standard documents 13321:1996 E and ISO 22412:2008 [49]. PDI is an indication of their quality with respect to the size distribution. Values of PDI greater than 0.7 indicate that the sample has a very broad size distribution and is probably not suitable for the DLS technique [50]. Based on DLS analysis, 90% of the nanoparticles in this study were in the range of 37.3 to 140.0 nm (Fig. 2, left) with a PDI of 0.247, showing that the nanochitin whiskers have a high dispersibility in aqueous solution. The zeta potential of 0.1% (w/v) nanochitin suspension was 26.67 mV with a data retention of 100%, showing effective enrichment amino groups in nanochitin (Fig. 2, right).

**Fig. 3:**
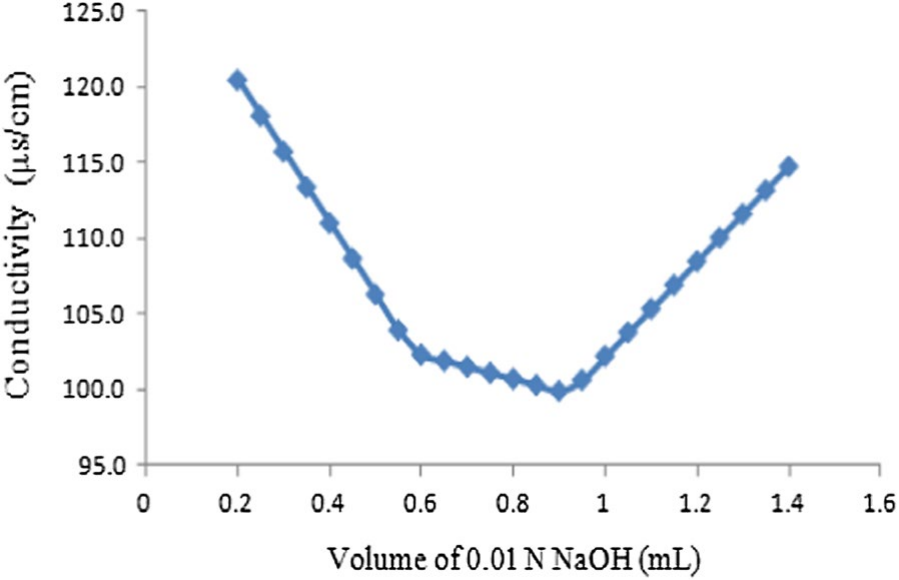
Conductometric titration curves for the determination of the amino group density of nanochitin

The amino group density of nanochitin was determined by conductometric titration. Figure 3 shows typical conductometric titration curves which had three sections. The initial linear decrease in conductivity was due to the neutralization of HCl and the lower mobility and molar conductivity of Na^+^ with respect to H^+^. After the first equivalence point, the decrease in conductivity slowed down. The middle section of the conductivity curve corresponded to the titration of the amino groups of nanochitin. At the second equivalence point, the conductivity started to increase upon additional NaOH because of excess of sodium and hydroxyl ions during the titration. Based on the calculation, the amino group density of nanochitin was 6.224 mol/kg.

### Promotive effect of nanochitin whisker to chemical aphicides on mortality of wheat aphid

Aphids are soft-bodied insects with piercing sucking mouthparts and feed on plant sap. *R. padi* is one of the major cereal pests of winter wheat and threatened wheat production in China. Control of *R. padi* has relied heavily on chemical insecticides [51, 52]. However, massive application of insecticides have resulted in insect resistance and raised serious human health and environmental concerns [53, 54].

Omethoate, imidacloprid and acetamiprid had significant toxic effects on wheat aphid, but they are not suitable to be used when wheat aphids outbreaks because of its short persistent period. For example, dilution of 40% Omethoate EC has displayed poor field control efficacy suggesting that wheat aphids had produced resistance to this insecticide [55]. Imidacloprid is a neonicotinoid insecticides as a new bionic pesticides. The insecticidal activity of imidacloprid is mainly acting on the acetylcholine receptor (AChR) in the nervous system of insects [54]. For future sustainable agricultural production, incorporation of nanotechnology within pesticides formulation will be a new tool to lower the indiscriminate use of conventional pesticides and for safe environmental applications [56].

Chitin and its derivatives have been proved lack of toxicity and not expected to harm people, pets, and wildlife, generally recognized as safe (GRAS) status as a food additive by the U.S. Food and Drug Administration [57–59]. Nanochitin material has been widely used in biomedical development with good biocompatibility, biodegradability, and non-cytotoxicity [60]. Zhao et al. [47] reported that nanochitin whiskers exhibited low toxicity at concentration lower than 50 µg/mL and had better cytocompatibility at 200 µg/mL concentration. Therefore, nanochitin would not cause damages to non-target populations.

**Fig. 4:**
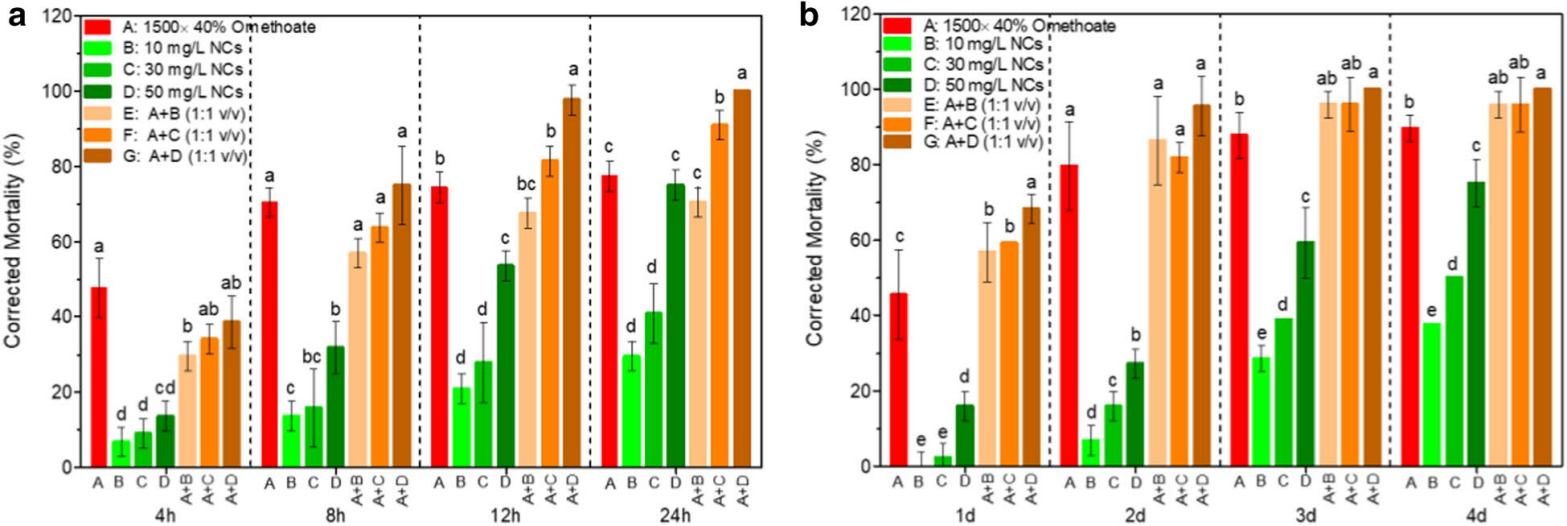
Corrected mortality of *R. padi* treated with different concentration nanochitin and the mixtures with Omethoate. Experiments were carried out by  **a** spraying contact, **b** continuous immersion. A-1500× dilution of 40% Omethoate (EC), B-10 mg/L NCs, C- 30 mg/L NCs, D-50 mg/L NCs. The mixtures of ×1500 dilution of 40% Omethoate (EC) with NCs were made by 1:1 in volume. Data are the means of three measurements. Error bars represent one standard deviation. Different letters above the bars represent significance at p < 0.05

To investigate the insecticidal activities and enhancement of nanochitin to chemical insecticides against wheat aphid, different concentration of NCs and its mixtures with dilutions of chemical pesticides, i.e. 1500× solution of 40% Omethoate (EC), 16,000× solution of 40% Acetamiprid (WG), .and 3000× of 10% of Imidacloprid (WP), were used for the treatments. *R. padi* was used as a model insect and tested by spraying-contact and continuous immersion methods respectively. We first tested the effectiveness of different concentrations of NCs and its mixtures with 1500× solution of 40% Omethoate (EC) at 1:1 by volume to obtain an optimal concentration of nanochitin in the mixture. The effects of nanochitin and its mixture with omethoate on aphid control were showed in Fig. 4 for spraying-contact and continuous immersion methods, respectively. Figure 4a showed that, at early stage, nanochitin alone by spraying contact method was unable to effectively control aphids, the corrected mortalities of aphids treated with nanochitin alone were much lower than 1500 × 40% Omethoate (EC). However, when mixed with 1500 × 40% Omethoate (EC), the corrected mortalities of aphids were all significantly enhanced after 4 h treatment and there was no significant difference among the treatments of the mixtures with nanochitin at concentration of 30 mg/L (A + C), 50 mg/L (A + D), and 1500 × 40% Omethoate (EC). After 8 h treatment, there is no significant difference in corrected mortality of aphids treated with all mixtures and 1500 × 40% Omethoate (EC). After 12 h treatment, the corrected mortalities of aphid treated with 50 mg/L of nanochitin alone reached 75.0% with a significant increase compared to the treatments with 10 mg/L and 30 mg/L. After 12 h treatment by the mixtures of nanochitin with 30 mg/L (A + C) and 50 mg/L (A + D), the corrected mortalities of aphids reached 81.4% and 97.7%. The control efficiency was dramatically improved by the mixture of 50 mg/L with 1500 × 40% Omethoate (EC), and there was a significant enhancement compared to other treatments, indicating that 50 mg/L was an optimal concentration to make an effective mixture with chemical pesticides. In application practice, the dosage of the chemical pesticide and nanochitin in the mixtures were all reduced to half due to half-by-half in volume mixing. Similarly, when continuous immersion method was employed (Fig. 4b), the corrected mortalities of the aphid, treatments with nanochitin alone at all three concentrations were significant lower than that of 1500 × 40% Omethoate (EC) dilution after 1 day treatment. But the corrected mortalities of the aphids treated with the mixtures were 56.8%, 59.1%, and 68.2% for the treatments of mixtures with 10 mg/L, 30 mg/L, 50 mg/L of nanochitin, respectively. There was a significant improvement in the mortality of aphid treated with the mixture of 50 mg/L nanochitin comparing with those of 1500 × 40% Omethoate (EC) and mixtures with 10 mg/L and 30 mg/L. After 2 days treatment, the corrected mortalities of aphids were 79.6%, 86.4%, 81.8%, and 95.5% for the treatments of 1500 × 40% Omethoate (EC), mixture with 10 mg/L, 30 mg/L, and 50 mg/L of nanochitin, respectively. There was no significant difference among these treatments other than those of treatments with nanochitin alone. We also found that a concentration of 50 mg/L of NCs had the highest effects on aphid’s control compared with 10 mg/L and 30 mg/L of NCs either applied alone or in the mixtures with 1500 × 40% Omethoate (EC). This result approved that nanochitin has a strong capability to increase the effectiveness of commonly used chemical pesticide, where as a mixture of chemical insecticide with nanochitin significantly decreases the usage of both components by half. It also offers a great potential to reduce the residue and pollution of agrichemicals in agriculture applications.

**Fig. 5:**
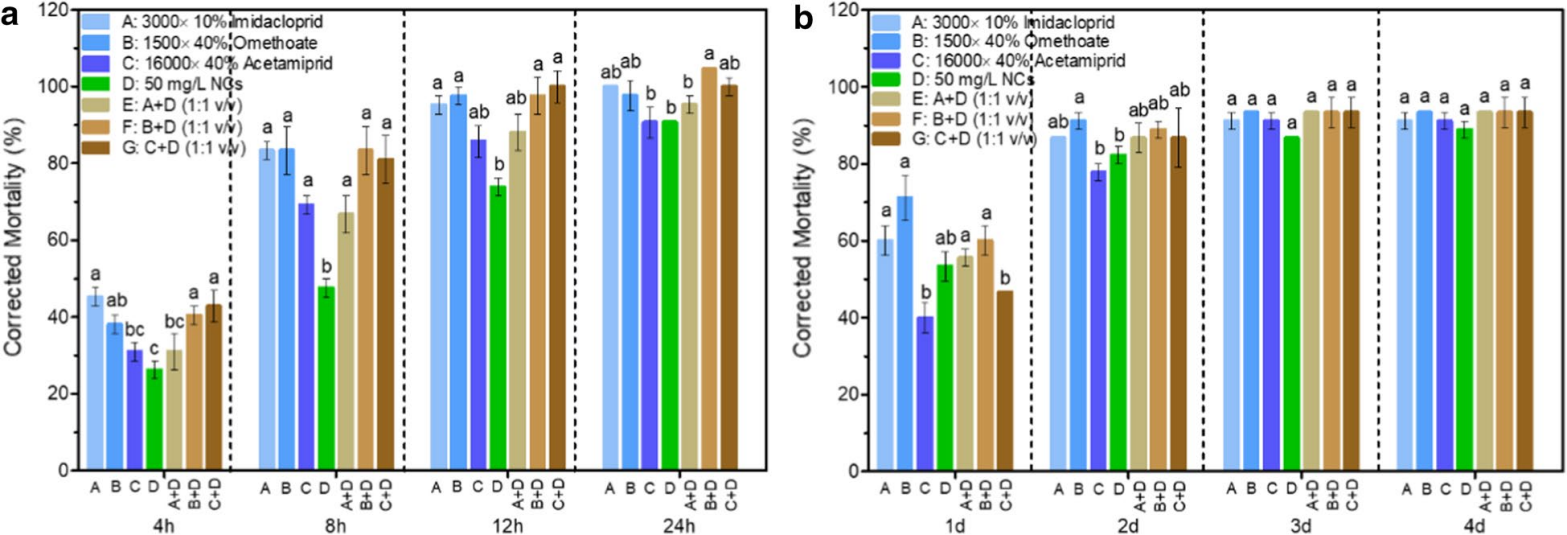
Comparison of mortality of *R. padi* treated with nanochitin and combinations with commonly used insecticides. Experiments were carried out by **a** spraying contact, **b** continuous immersion method. A: ×3000 dilution of 10% Imidacloprid (WP), **b** ×1500 dilution of 40% Omethoate (EC), C: ×16,000 dilution of 40% Acetamiprid (WP), D: 50 mg/L NCs. The mixtures were made by volume of two components at ratio of 1:1. Data are the means of three measurements. Error bars represent one standard deviation. Different letters above the bars represent significance at p < 0.05

More synergistic effects of nanochitin on commonly used pesticide on insecticidal efficacy were investigated in a separate experiment. Three commonly used pesticides including 3000× dilution of 10% Imidacloprid (WP), 16,000× dilution of 40% Acetamiprid (WG), and 1500× dilution of 40% Omethoate (EC, 1500×), as well as their mixtures with 50 mg/L of NCs (1:1 by volume) were used to evaluate the improvement of insecticidal efficacy in aphid control either by spraying-contact (Fig. 5a) or continuous immersion method (Fig. 5b). Figure 5a showed that at first 4 h treatment by spraying contact method, 50 mg/L of NCs has a low efficiency in aphid control comparing with three chemical insecticides. However, when treated with the mixtures of nanochitin with dilutions of Omethoate and Acetamiprid, the mortality of aphid significantly increased compared to the treatment of nanochitin alone. After 8 h treatment, the corrected mortalities of aphids in treatments with the mixtures were all significantly enhanced. After 12 h treatment, the corrected mortalities reached 80% or above in all treatments. The aphids treated with continuous immersion method (Fig. 5b), the control efficacy of nanochitin was almost the same as other treatments after 1 days. After 2 days treatment, the corrected mortalities of aphids were all great than 80% and there was no significantly differences among the treatments of nanochitin and its mixtures with the chemical pesticide dilutions. The usages of pesticides in the mixtures with 50 mg/L of nanochitin were all reduced by 50%, demonstrating a great significance in aphids control and benefit to environmental sustainability as well.

**Fig. 6:**
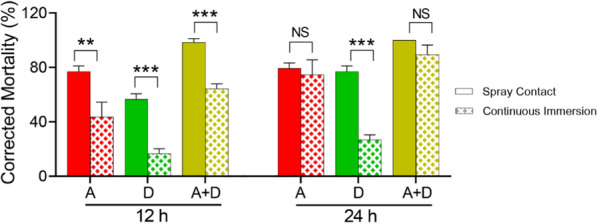
Comparison of corrected mortality for *R. padi* treated by different treatment method. A-1500× dilution of 40% Omethoate (EC), D- 50 mg/L of nanochitin suspension, A + D: the mixture of A and D by 1:1 in volume. NS: no significant difference at level p < 0.05, **: p < 0.05; ***: p < 0.01, one-way ANOVA

To compare the corrected mortality for *R. padi* between different treatment methods, the data of treatments of 1500× dilution of 40% Omethoate (EC) and 50 mg/L of nanochitin in different treatment methods were selected and analyzed statistically. The results showed that spray contact method was more effective treatment method (Fig. 6). There was a significant difference between spray contact and continuous immerse methods after 12 h treatment. For the treatment of 1500× dilution of 40% Omethoate (EC), the significant difference between two methods was at significance level of p < 0.05, however, the significant difference for the treatments of 50 mg/L alone and its mixture with 1500× dilution of 40% Omethoate (EC) was at the significance level of p < 0.01. After 24 h treatment, no significant difference for 1500× dilution of 40% Omethoate (EC) and its mixture with 50 mg/L f nanochitin between these two treatment methods. But spray contact for 50 mg/L of nanochitin alone was more effective in aphid control, the mortality of aphid between these two methods has a significant difference at significance level of p < 0.01.

Overall, nanochitin alone in a range of 10−50 mg/L has a limited control efficiency in aphid control. When associated with chemical insecticides nanochitin significantly improved the insecticidal activity and greatly reduced chemical pesticide use. The synergistic effects of nanochitin whisker to chemical pesticides on the aphids control might be attributed to its small size and high surface area. It has been reported that α-chitin nanocrystals from shrimp shells exhibited exceptionally high surface area near 350 m^2^/g [35], giving a great opportunity to associate with chemical pesticide. The results in this study also make a great agreement with previous reports [2, 43, 44]. Moreover, an interesting phenomenon was observed during feeding with NCs (Additional file 1: Fig S1). Aphids stopped suckling after inoculated on the treated leaves (Additional file 1: Fig S1a). Some aphids moved away from the leaves (Additional file 1: Fig S1b) and some died (Additional file 1: Fig S1c) after 4 h treatment. It might indicate that nanochitin may have another effect on aphid behavior as an antifeedant. More study will be conducted in near future.

### Uptake and translocation of nanochitin whisker inside aphids

The toxicological mechanism of nanochitin whisker in aphid control and promotable activity to chemical insecticides is still unknown. Nonetheless, labelling of biomolecules by fluorescent probe has become an important tool to detect target analytes in the fields of biology and medicine. FITC is an organic dye with excitation and emission spectrum peak wavelength at approximately 495 nm and 519 nm respectively, giving it a green color examined by a fluorescent microscope. FITC and its derivatives are widely applied fluorescence reagents with high absorptivity, excellent fluorescence quantum yield and good water solubility [61]. Hassan et al.[62] demonstrated that primary amine group of D-glucosamine residue can be labelled by isothiocyanate group of FITC and used to monitor cellular localization of biomaterials in a biological system. Nanochitin particles, on the other hand, carry certain amount of free amino group on surface of the particle after acidic hydrolysis. Therefore, nanochitin could be labelled with FITC by conveniently reacting its amino group with isothiocyanate group of FITC. FNC emits intense fluorescence in the non-polar solvent and can be used to track the transportation pathway and acting location of nanochitin inside aphid.

To illustrate the transportation pathway of nanochitin in aphid, FNC was applied to wheat seedling, then aphids were inoculated on the leaves of treated plants. DI water served as a control for this test. The experiment was carried out by continuous immersion method. The transportation pathway and acting location of FNC particles inside aphid was examined by laser confocal scanning microscope (ZEISS LSM 700, Carl Zeiss Co., Germany) or fluorescent microscopy using Leica DM IL-LED microscope (Leica DM IL-LED, Inverted Laboratory Microscopy with LED Illumination, Germany).

**Fig. 7:**
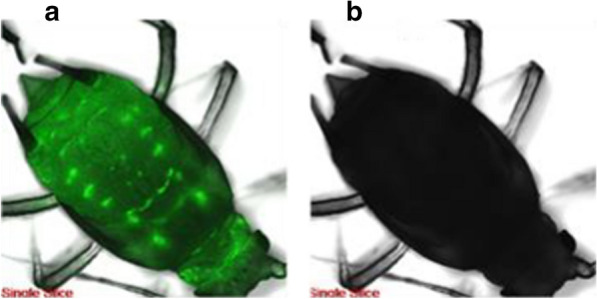
Fluorescent microscopy of FITC-labelled nanochitin absorbed by wheat aphids. The fluorescence was detected with laser confocal scanning microscope. **a** demonstration of uptake and translocation of FITC-labelled nanochitin inside an alive aphid after 12 h treatment. **b** control

Figure 7 simply demonstrated the absorption and distribution of fluorescence-labelled nanochitin in aphids. The path of the absorption and translocation of the FNC over time was showed in Additional file 2: Fig S2. Theoretically, at an early stage of feeding, FNC was absorbed along with plant fluid from mouth and digestive system and distributed to the back of the body. From Additional file 2: Fig. S2 we can see that, the FNC was distributed to all parts of the body after 24 h (Additional file 2: Fig. S2-A). Over the time of absorption and distribution, FNC was translocated and accumulated in certain parts of the body (Additional file 2: Fig. S2-B). After 96 h treatment, the FNC was mostly accumulated at the cornicles area and might be emitted to outside of the body with decay of the fluorescent (Additional file 2: Fig. S2-C).

The phenomena observed in this study indicated that nanochitin can be distributed from its digestive system and relocated to other tissues. Nanochitin might continue to accumulate and remain in certain parts of the body over the time and caused significant death of aphids. It closely corresponded to the results showed in Fig. 4 that, after 12 h treatment, the corrected mortality of aphids treated with 50 mg/L of nanochitin alone and its mixtures with dilutions of chemical pesticides reached 80% or above, meaning that most of treated aphids dead. In our previous study, we found that NCs significantly promoted abamectin (Am) water dispersity, thermal stability, and photostability, effectively reduced Am dosage in insect pest control [43]. Am is a biogenic pesticide derived from the bacterium *Streptomyces avermitilis.* Its water insolubility and light-degradability greatly influence its applications in plant protection practice. Nanochitin could not only protect Am from hush environmental stability but also improve its insecticidal activity significantly when two compounds associated each other. The hydrophilic property of nanochitin and the lipophilic property of Am drove a conjugation formation but disassociated from two components at different pH [63]. The results in this study also showed a strong evidence that nanochitin synergistically enhanced insecticidal activity of systemic organophosphorus and neonicotinoid insecticides. When mixed with these chemicals, the use of the chemical pesticides could be significantly reduced. Moreover, foliar application of nanochitin and its combination with chemical pesticides was most effective application method to control the damage of aphids in a short period of time.

### Acute oral and dermal toxicity in mice

 Toxic potential of materials at nano level have drawn a lot of attention recently. Despite its potential in generating toxicity to biological systems and the environment, materials in nanosize may perform exceptional feats of conductivity and optical sensitivity. To evaluate the toxicity of nanochitin, standard biological assay was conducted using SD rat for acute oral and dermal toxicity determination.

**Table 1:** Acute oral toxicity of NCs to mice

Gender	Dose (mg/kg)	Number of Mice	Body Weight (`x ± SD) (g)			Number of	Death Rate	LD_50_ at P < 0.05
			0d	7d	14d	Death	(%)	(mg/kg)
Male	5000	5	195.8±4.7	212.8 ± 4.8	233.6 ± 4.8	0	0	> 5000
Female	5000	5	193.6 ± 5.1	207.2 ± 5.1	224.8 ± 5.3	0	0	> 5000

**Table 2:** Acute dermal toxicity of NCs to mice

Gender	Dose (mg/kg)	Number of Mice	Body Weight (`x± SD) (g)			Number of	Death Rate	LD_50_ at P < 0.05
			0d	7d	14d	Death	(%)	(mg/kg)
Male	2000	5	223.0±2.7	214.4 ± 3.3	263.8 ± 3.3	0	0	> 2000
Female	2000	5	220.6 ± 3.6	235.0 ± 4.0	254.0 ± 3.8	0	0	> 2000

The toxicity results of nanochitin to SD rat showed in Tables 1 and 2. The original NCs (stock suspension, 0.3% (w/v)) was used as biological material. The acute oral toxicity LD50 is great than 5000 mg/kg BW in male and female (Table 1), acute dermal toxicity LD50 is great than 2000 mg/kg BW (Table 2)

Many studies have demonstrated that chitin and chitosan nanoparticles were suitable materials for biomedical applications due to its high biocompatibility, non-toxicity [47, 64]. Azuma et al. [64] reported that nanochitin whisker had a low toxicity to non-cancer cells but had obvious cytotoxicity to many kinds of cancer cells. According to Zhao et al.[47], chitin nano-whisker had good biocompatibility below the concentration of 2.5 mg/mL. They also found cell viabilities of mouse bone marrow mesenchymal stem cells (mBMSCs) and rat osteosarcoma cells (UMR-106) treated with nanochitin at a concentration of 200 µg/mL reached 96.2% and 99.8% respectively. Solairaj et al. [65] reported that metal composited with chitin nanoparticles had stronger cytotoxicity to human breast cancer cells (MCF-7) but non-toxic to healthy cells. Our results showed that the acute oral and dermal toxicity LD50 were nontoxic to the tested mice at a concentration of 0.3% (w/v, 3,000 µg/mL), which makes a good agreement with previous study done by Zhao et al. [47]. However, the synergistic effects of nanochitin composited with chemical pesticides on oral and dermal toxicity in mice need to be further investigated

## Conclusions

Cationic chitin nanoparticles synthesized by acidic hydrolysis were rod-like whiskers. The size of the whisker was in a range of 50–150 nm in length and 15–50 nm in width examined by TEM and FE-SEM. The amino group density of nanochitin whisker in aqueous solution was 6.224 mol/kg. Nanochitin whiskers can be easily absorbed and transported in digestive system of insects through sucking by piercing-sucking mouthparts and showed insecticidal activity against piercing-sucking mouthparts insects. By treated with 30–50 mg/L of NCs, the corrected mortality of wheat aphids reached 80% or above after 12 h treatment. When mixed with dilution of conventional pesticides, the corrected mortality of aphid significantly enhanced up to 95% or above. Acute oral and dermal toxicity assays (for LD_50_) were performed using doses of 5000 mg/kg BW and 2000 mg/kg BW, the results showed that nanochitin was non-toxic to the model animal SD rat. As a biocompatible and biodegradable natural polymer, nanochitin formulated pesticide system will have a great potential for agricultural applications aiming at enhancement of insecticidal activity, significant reduction of pesticides use in pests control. However, the dermal and oral toxicity for mice at higher dosages, environmental risk assessment, and toxicological mechanism of nanochitin whiskers from molecular level will be further investigated.

## Supplementary Information

**Additional file 1.** Effect of nanochitin on aphid behavior and activity after 4 h treatment.

**Additional file 2.** Fluorescent microscopy of FITC-labelled nanochitin absorbed by wheat aphids.
