# Supplementary material for: Nanochitin whisker enhances insecticidal activity of chemical pesticide for pest insect control and toxicity
Source: J Nanobiotechnology. 2021 Feb 16;19:49. doi: 10.1186/s12951-021-00792-w (PMC7885609; doi:10.1186/s12951-021-00792-w)
Supplement: Supplementary file 1 — Additional file 1. Effect of nanochitin on aphid behavior and activity after 4 h treatment. [file 12951_2021_792_MOESM1_ESM.pdf]

Nanochitin whisker enhances insecticidal activity of chemical pesticide for pest insect control and toxicity

Zhenya Li[1,3], Hezhong Wang[2,3*], Shiheng An[1], Xinming Yin[1*]

[1]Department of Entomology, Henan Agriculture University, Zhengzhou, 450002, China

[2]Department of Pesticide Science, Henan Agricultural University, Zhengzhou, 450002, China

[3]NanoAgro Center, Henan Agricultural University, Zhengzhou 450002, China

* Correspondence: Hezhong Wang: hezhongw@126.com; ORCID: 0000-0002-9281-8533;

Xinming Yin: xmyin@henau.edu.cn

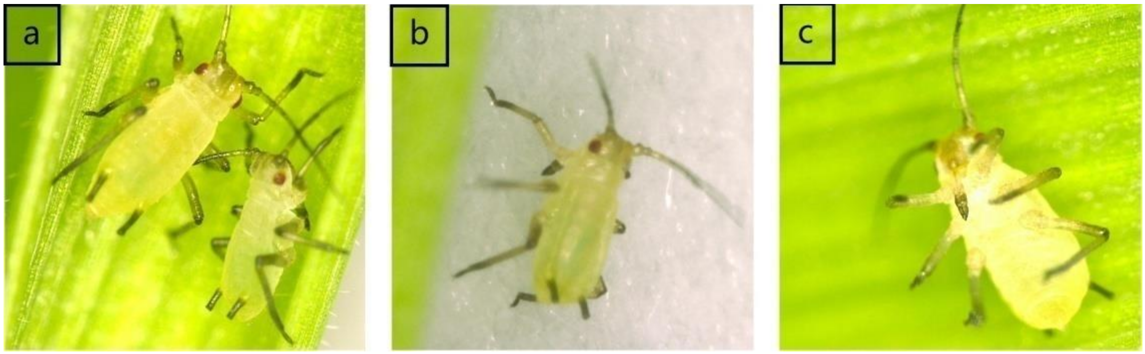

Fig. S1 Effect of nanochitin on aphid behavior and activity after 4 h treatment. The treatment was carried out by spraying contact method with 50 mg/L of nanochitin whisker suspension. Aphids stopped suckling after inoculated on the treated leaves (a). Some aphids moved away from the leaves (b) and some died (d) after 4 h treatment.
